# Metastasis-related long non-coding RNAs AL359220.1, SH3BP5-AS1 and ZF-AS1 are significant for prognostic assessment of lung adenocarcinoma

**DOI:** 10.18632/aging.204923

**Published:** 2023-08-10

**Authors:** Jianjun Tan, Weilin Mao, Shuzi Long, Tao Zhang

**Affiliations:** 1 Department of Oncology, The First Affiliated Hospital of Chongqing Medical University, Chongqing 400016, China; 2 Department of Oncology, Three Gorges Hospital of Chongqing University, Chongqing 404000, China

**Keywords:** lung adenocarcinoma, metastasis, long non-coding RNA, risk model

## Abstract

Background: Metastasis of lung adenocarcinoma (LUAD) severely worsens prognosis. Genetic alteration in the tumor microenvironment (TME) is closely associated with metastasis and other malignant biological properties of LUAD. In this study, we establish a metastasis-related risk model to accurately predict LUAD prognosis.

Methods: RNA-sequencing profiles and clinical data of LUAD patients including 503 tumor tissues and 54 adjacent normal tissues were collected in TCGA database. Additionally, the paired specimens from 156 LUAD patients were obtained in a single center. The metastatic relevance and clinical significance of metastasis-related long non-coding RNA (MRLNRs) was validated by series of *in vitro* experiments including western blotting, qPCR and transwell assays.

Results: Six MRLNRs were significantly correlated to prognoses of LUAD patients, of which AL359220.1, SH3BP5-AS1 and ZF-AS1 were further used to establish a metastasis-related risk scoring model (MRRS) due to the close associations with overall survival of LUAD patients. According to the MRRS, patients with higher scores in the high-risk group obtained poorer prognoses and survival outcomes. ZFAS1 expressed highly in tumor tissues and showed the inverse results compared to SH3BP5-AS1 and AL359220.1. In addition, the high expression of ZFAS1 was prominently correlated to the more advanced T-stage and distant metastasis. The reduction of ZFAS1 induced by siRNAs dramatically diminished the migration and invasion abilities of LUAD cells.

Conclusions: In the present research, we elucidate the metastatic relevance and clinical significance of AL359220.1, SH3BP5-AS1 and ZF-AS1 in LUAD. Moreover, MRRS provide a promising assessing model for clinical decision making and prognosis of LUAD.

## INTRODUCTION

Lung adenocarcinoma (LUAD) is a common pulmonary malignancy with a poor prognosis. With the higher proportion than squamous cell carcinoma and large-cell carcinoma, lung adenocarcinoma (LUAD) accounts for approximately 60% of non-small cell lung cancer which is the majority of lung cancer [1]. Lung cancer remains the leading cause of cancer-related deaths worldwide, with a significant impact on public health. It is estimated that there are approximately 2.09 million new cases of lung cancer and 1.76 million deaths attributed to the disease each year. Due to the lack of effective and sensitive diagnostic methods in the early stages, LUAD is often examined when original tumor cells metastasize to the brain, bones, and respiratory system, provoking the consistently high mortality [2–5]. Although a chain of new therapeutic strategies including chemotherapy, targeted therapy and immunotherapy have been approved in clinic, the long-term outcomes for metastatic LUAD proved unsatisfactory [6, 7]. Importantly, specific mechanisms underlying metastasis of LUAD and its association with the tumor microenvironment (TME) remain incompletely understood. Therefore, the pathogenesis and metastatic mechanism of LUAD must be determined for early evaluation and treatment.

It has been well appreciated that TME, providing the crucial intercellular platform, closely affects tumorigenesis, progression and long-term prognosis by modulating the functional and genetic alteration in cell subpopulations [8–10]. In the last decades, overwhelming evidence suggests that differentially expressed gene (DEG) in the TME are not only promising biomarkers for prognostic evaluation including metastasis assessment, but also act as effective targets for cancer treatment [11–13]. A series of metastasis-related long non-coding RNA (MRLNRs) have been identified to regulate the metastatic processes of various tumors. The high expression of lncR-MCF2L-AS1A in breast tumor cells promotes metastasis by increasing YAP transcription [14]. In addition, Dong et al. found a positively modulating relationship between lncR-TRIM28-14 and COL4A1 in the peritoneal metastatic tissues of gastric cancer, indicating the potential role of lncR-TRIM28-14 in metastatic evaluation and strategy [15]. Nevertheless, the pivotal role of lncRNAs in the metastatic process of LUAD remain incompletely understood.

In this study, we will identify more promising MRLNRs to construct an accurate risk model for prognostic evaluation and targeted treatment and how MRLNRs regulate the metastasis of LUAD. These findings will provide a novel perspective for further research in the realm of lncRNA-modulated LUAD metastasis.

## RESULTS

### Acquisition of MRLNRs

**Figure 1:**
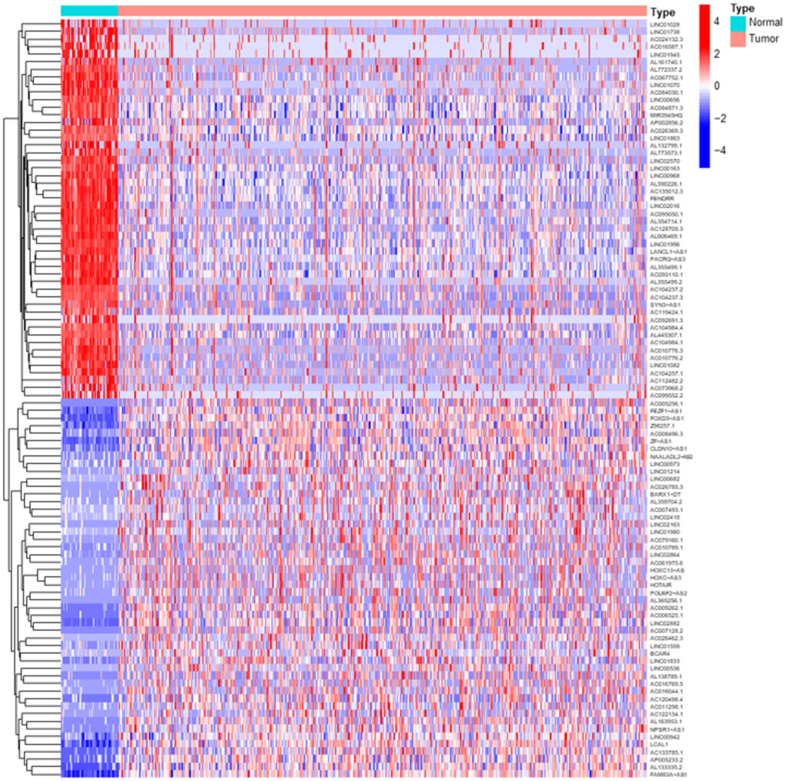
**Differentially expressed LUAD lncRNAs.** The heatmap showed the top 50 upregulated and downregulated DElncRs between LUAD tumor tissues and normal tissues. The blue parts represent downregulated lncRNAs and the red parts represent the upregulated lncRNAs.

Transcriptome RNA-sequencing data and clinical data were downloaded from TCGA database. As a standard of P< 0.05 and |log2 fold change| > 1, we screened 3278 DElncRs, of which 2426 were upregulated and 852 were downregulated, and the top 50 upregulated and downregulated DElncRs were illustrated in Figure 1A. Following that, lncRNAs and mRNAs data were extracted from transcriptome data. We screened 44 MRGs in TOMIDA_METASTASIS_UP M17830 and TOMIDA_METASTASIS_DN M2583 of Molecular Signatures Database, of which 339 lncRNAs were identified to be the MRLNRs through correlation analysis.

### The correlation between prognosis of LUAD patients and MRLNRs

**Figure 2:**
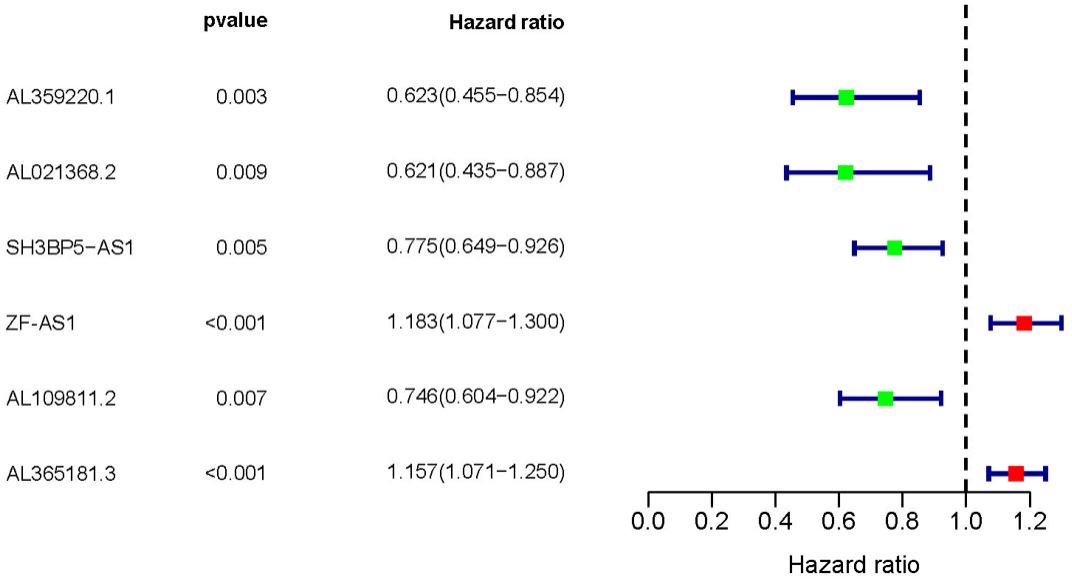
**Survival-related MRLNRs.** Forest plot illustrated the prognosis values of sMRLNRs (AL359220.1, AL021368.2, ZF-AS1, SH3BP5-AS1, AL109811.2 and AL365181.3).

Based on COX regression analysis, we then verified 6 MRLNRs which were associated with prognosis of LUAD patients, including AL359220.1, AL021368.2, SH3BP5-AS1, AL590666.2, AL109811.2 and ZF-AS1 (p<0.01). The relationships between these sMRLNRs and LUAD prognoses were clearly illustrated in the forest plot (Figure 2).

### Prognostic features of the high-risk group and the low-risk group

**Figure 3:**
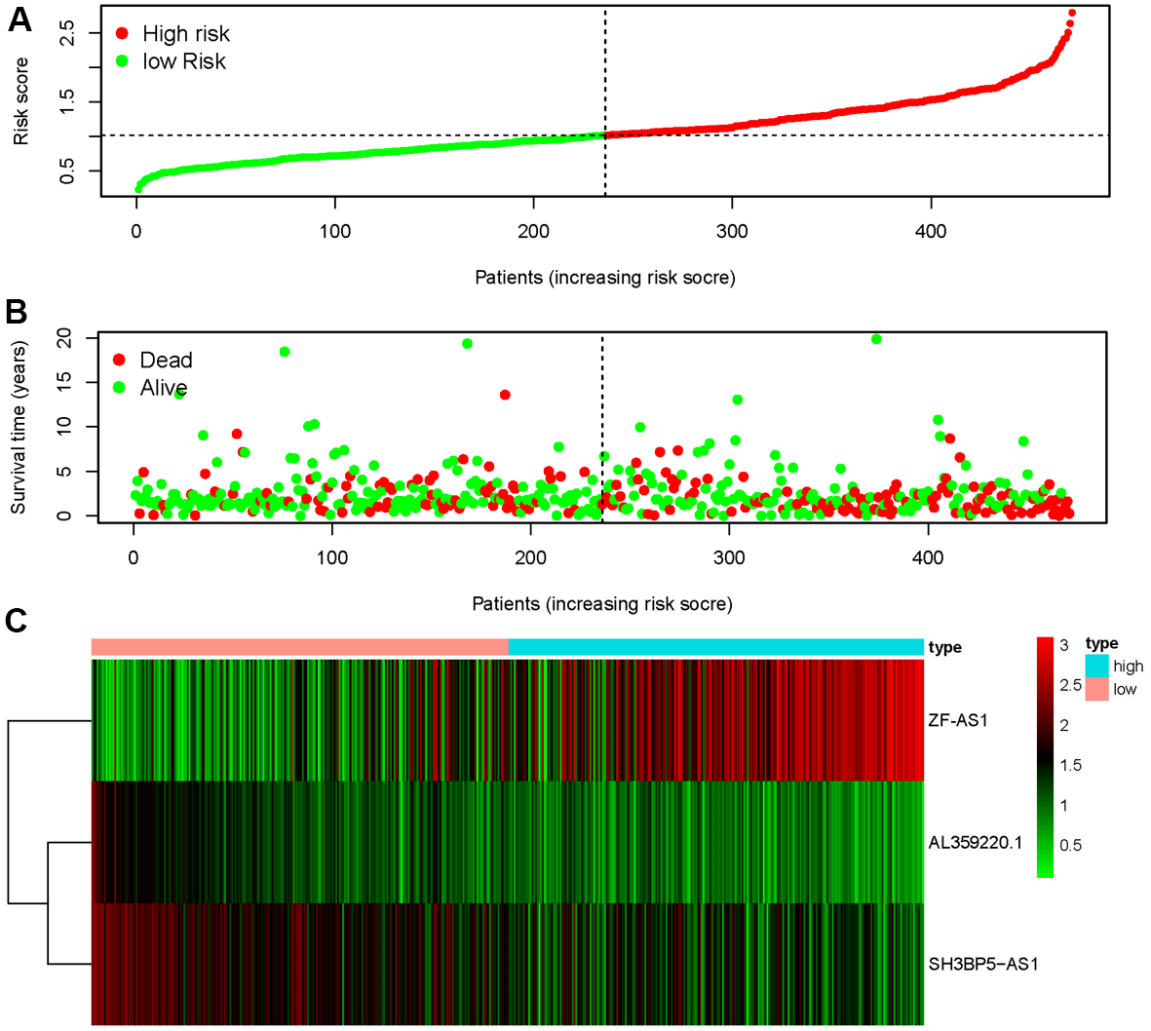
**Metastasis-related risk score model (MRRS) was established based on sMRLNRs.** The risk score distribution in low-risk group and high-risk group of LUAD patients (**A**). Survival status between LUAD patients’ high-risk group and low-risk group (**B**). The heatmap of expression levels of contained sMRLNRs (**C**).

**Figure 4:**
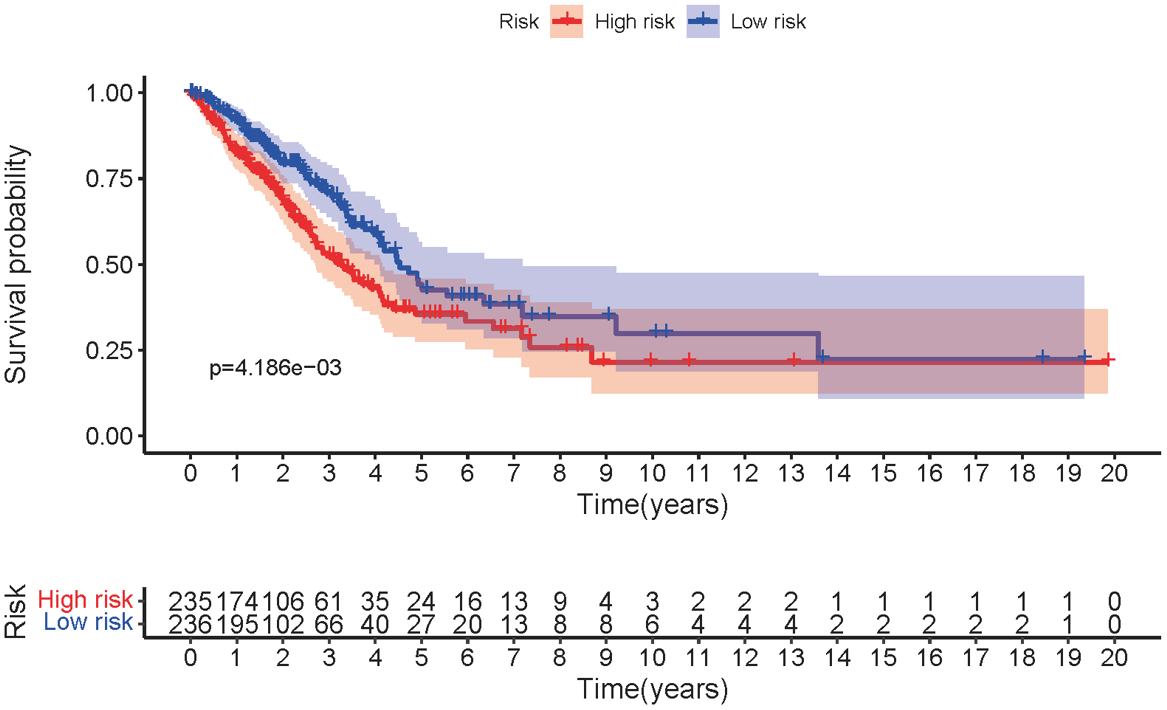
**The survival curve of MRRS.** Kaplan-Meier survival curve of survival probability in low-risk and high-risk groups of LUAD patients.

The three sMRLNRs (AL359220.1, SH3BP5-AS1 and ZF-AS1) among the 6 sMRLNRs were selected by multivariate COX analysis (*P*<0.05)., and were used to establish the MRRS, by which the LUAD patients were divided into the high-risk group and the low-risk group (Figure 3A). As illustrated in the Figure 3B, the mortality rate constantly increased with the higher risk score. With the increase of the risk score, the expression levels of ZF-AS1 were increased, while SH3BP5-AS1 and AL359220.1 were decreased (Figure 3C). Furthermore, the survival curve of the high-risk group was significantly lower than that of the low-risk group (Figure 4).

### The relationships between MRRS and clinical features and the regulation network of sMRLNRs

**Figure 5:**
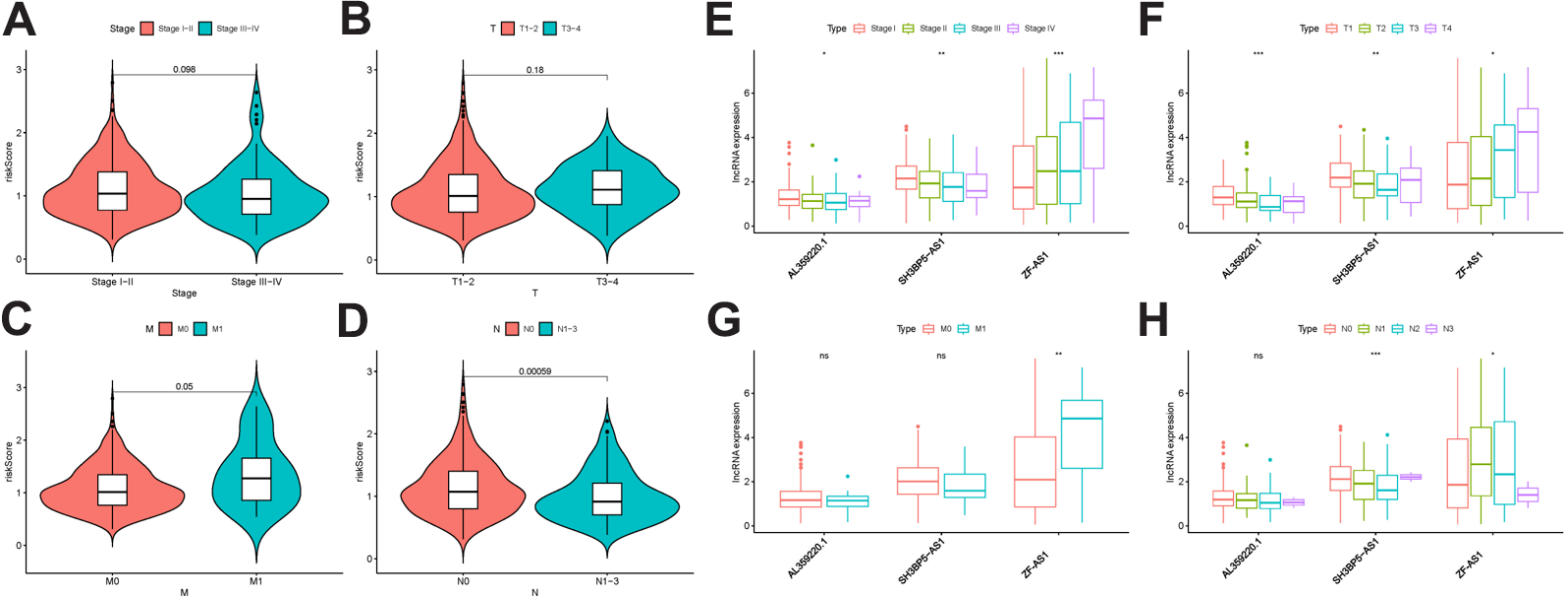
**The relationship between the risk score and clinical features.** Relationships between MRRS and stage (**A**), T- stage (**B**), M stage (**C**) and N- stage (**D**). Relationships between sMRLNRs and stage (**E**), T-stage (**F**), M-stage (**G**) and N-stage (**H**).

To further explore the relevance of the sMRLNRs and clinical features of LUAD, we analyzed the correlation of MRRS and the clinical and demographic characteristics including stage, T-stage and N-stage and M-stage (Figure 5A–5H). We found that the MRRS was significantly correlated with N-stage and M-stage (Figure 5C, 5D). We found the expression levels of SH3BP5-AS1 and AL359220.1 were lower in LUAD patients with the more advanced stage and T-stage (Figure 5E, 5F). Furthermore, the expression of SH3BP5-AS1 was correlated with early N-stage. Besides, the expression of ZF-AS1 was higher with the more advanced stage, T-stage, N-stage and M-stage (Figure 5E–5H). We further displayed the independent prognostic factor analysis, the results showed stage, T-stage, N-stage, M-stage and risk score were prominently correlated with OS in univariate analysis (*P*<0.05). However, only risk score illustrated as an independent risk factor in the multivariate analysis (Table 1). The ROC curves could represent the accuracy of the MRRS.

**Table 1:** Univariate and multivariate COX analysis of LUAD patients.

	**Univariate analysis**				**Multivariate analysis**			
**Variables**	**HR**	**HR 95% low**	**HR 95% high**	**P value**	**HR**	**HR 95% low**	**HR 95% high**	**P value**
**Age**	1.009472	0.993732	1.025461	0.239667	1.009939	0.993923	1.026213	0.225263
**Gender**	1.148882	0.848326	1.555922	0.369753	0.902694	0.653771	1.246394	0.534001
**Stage**	1.600743	1.385203	1.849821	1.81e-10	1.444730	0.991066	2.106060	0.055709
**T-stage**	1.543313	1.285402	1.852972	3.30e-06	1.150620	0.933031	1.418958	0.189570
**M-stage**	1.966135	1.114580	3.468287	0.019566	0.652789	0.247017	1.725119	0.389686
**N-stage**	1.632947	1.371507	1.944224	3.61e-08	1.135570	0.816321	1.579672	0.450306
**Risk score**	2.552644	1.806082	3.607805	1.10e-07	2.052003	1.417997	2.969481	0.000137

Note: HR, Hazard Ratio.

**Figure 6:**
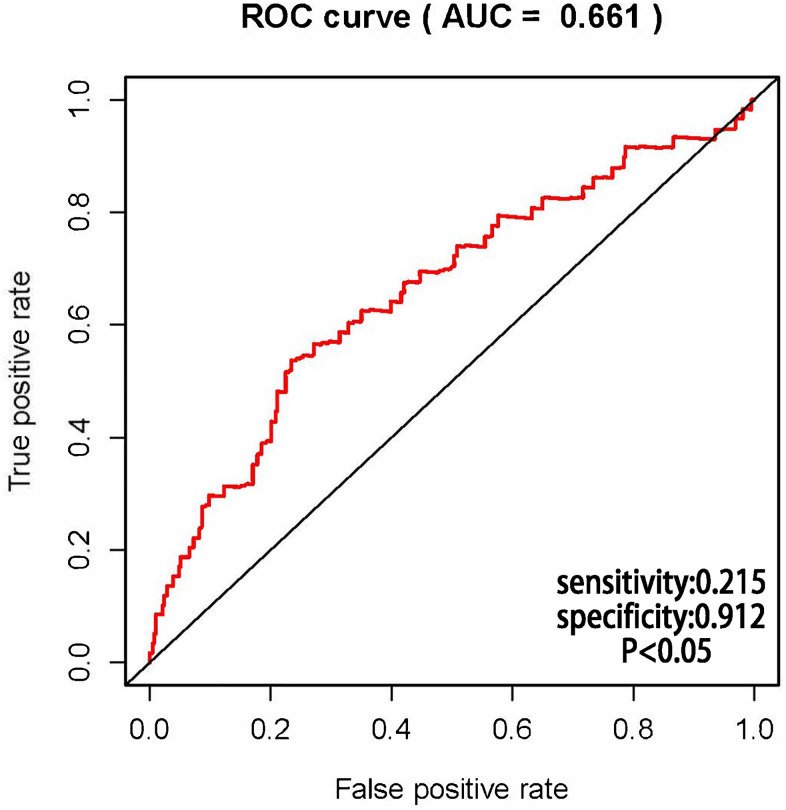
**Receiver operating characteristic (ROC) curve.** ROC curves demonstrated the prognostic accuracy of MRRS. The AUC of risk score was 0.661.

**Figure 7:**
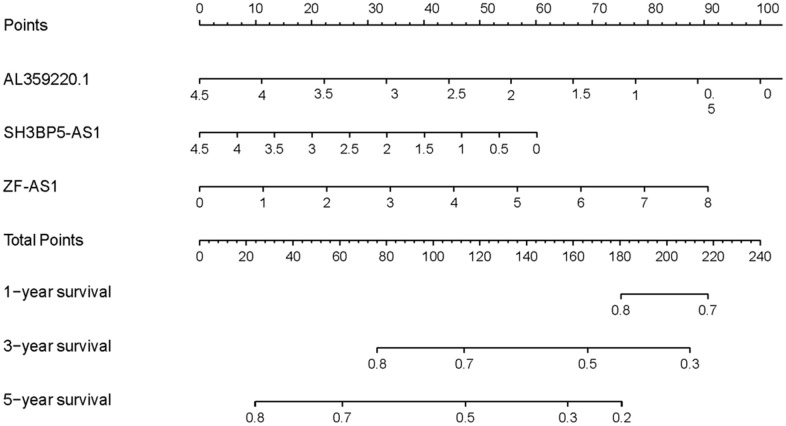
**Nomogram of MRRS.** Nomogram was drawn to predict LUAD patients’ 1-, 3-, and 5-year survival probability by evaluating the expression of sMRLNRs.

**Figure 8:**
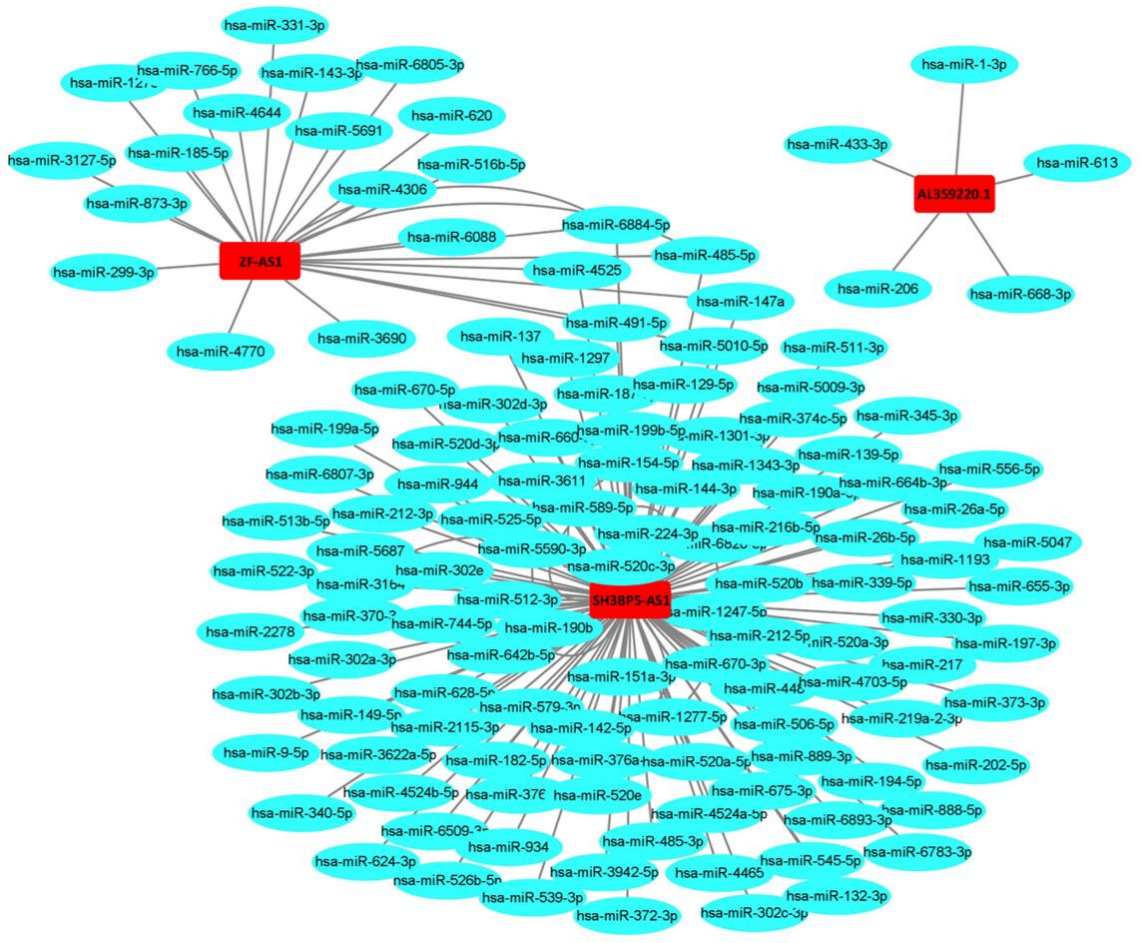
**The relationships of the sMRLNRs and target miRNAs.** The sMRLNRs and target miRNAs regulatory network. Red parts represent sMRLNRs; blue parts represent miRNAs.

The AUCs of risk score was 0.661, sensitivity:0.215 and specificity:0.912 (Figure 6). These results suggested that the MRRS can be regarded as an accuracy predict model for LUAD. Next, the MRRS was integrated into a nomogram for predicting 1-, 3-, and 5-year survival rates of LUAD patients (Figure 7). Based on the nomogram scores, the 1-, 3-, and 5-year survival rates of LUAD patients could be well predicted by their nomogram scores. To further find the regulatory relationships of sMRLNRs and their target microRNAs, we selected the microRNAs of Starbase database and the results were demonstrated in regulatory network (Figure 8).

### LncR-ZFAS1 showed significant relevance to metastasis

**Figure 9:**
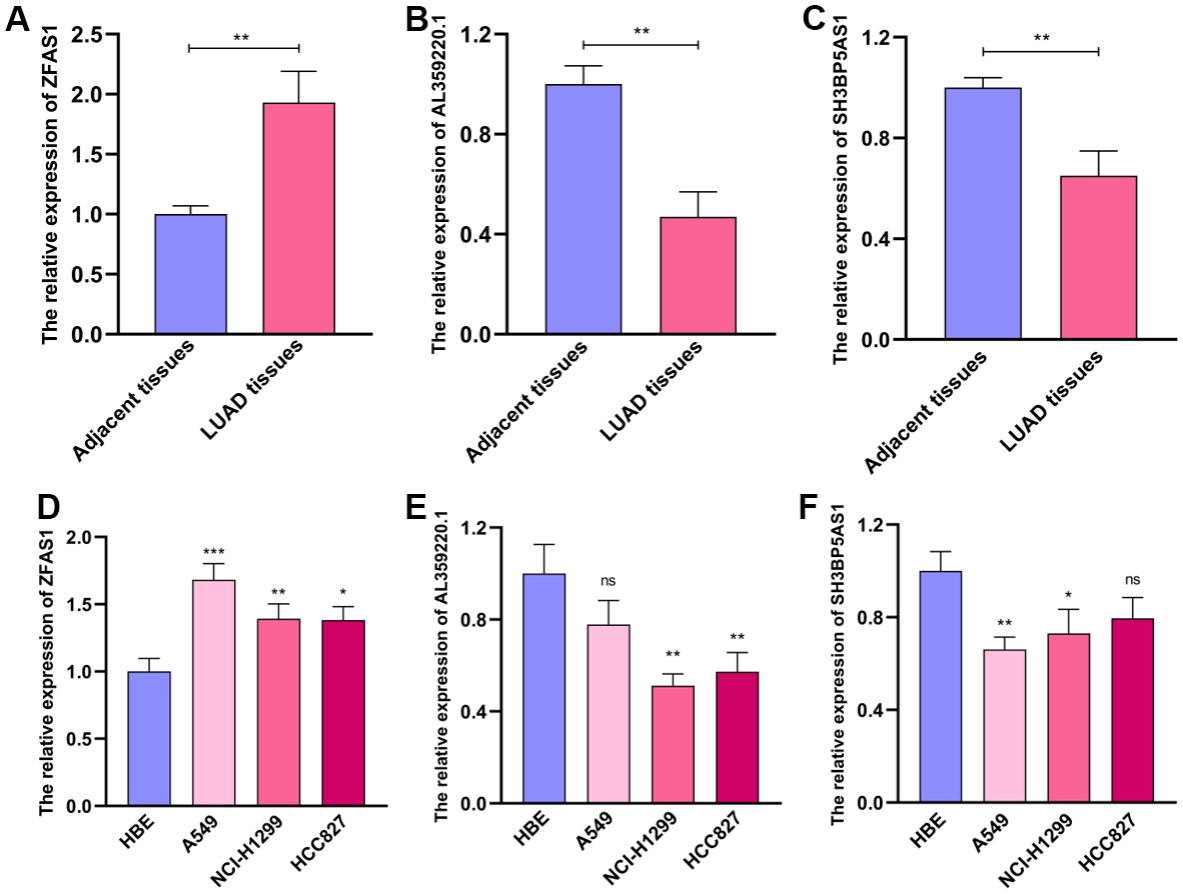
**The expression levels of AL359220.1, ZFAS1 and SH3BP5-AS1 in cell lines and LUAD tissues.** The qPCR results of the expression levels of ZFAS1, AL359220.1 and SH3BP5-AS1 in LUAD tissues and adjacent tissues (**A**–**C**) and LUAD cell lines (A549, HCC827 and NCI-H1299) and human bronchial epithelial cell (HBE). (**D**–**F**) ZFAS1 highly expressed in LUAD tissues and LUAD cell lines than adjacent tissues and HBE cell. The expression of AL359220.1 and SH3BP5-AS1 in adjacent tissues and HBE cell were higher than that in LUAD tissues and LUAD cell lines.

Then, we examined the levels of these sMRLNRs which were used to establish the MRRS in various LUAD tissues and cell lines, and found that ZFAS1 expressed highly in tumor tissues and showed the inverse results compared to SH3BP5-AS1 and AL359220.1 (Figure 9A–9C). Consistently, the expression of ZFAS1 in LUAD cell lines (A549, NCI-H1299 and HCC827) was dramatically higher than that in normal bronchial epithelial cell line, while SH3BP5-AS1 and AL359220.1 expressed increasingly in HBE cell line (Figure 9D–9F).

In addition, we further investigated the clinical relevance of ZFAS1. All the LUAD patients were divided into the ZFAS1-high-expression group and ZFAS1-low-expression group, followed by the analyses of their associations with various clinicopathologic features. As illustrated in Table 2, ZFAS1 showed prominent correlation to T-stage and metastatic status, which was consistent with the finding in Figure 5C, 5D.

**Table 2:** The associations between ZFAS1 and clinicopathologic features of LUAD patients.

**Parameter**	**N**	**The ZFAS1 level**		***P* value**
		**Low**	**High**	
**Gender**				0.623
**Male**	97	42	55	
**Female**	59	28	31	
**Age (year)**				0.746
**<65**	89	43	46	
**≥65**	67	35	32	
**T stage**				0.002
**1**	41	31	10	
**2**	85	34	51	
**3**	26	12	14	
**4**	4	1	3	
**Metastatic status**				0.017
**Positive**	13	2	11	
**Negative**	143	76	67	

### LncR-ZFAS1 strikingly promoted migration and invasion abilities of LUAD cell lines

**Figure 10:**
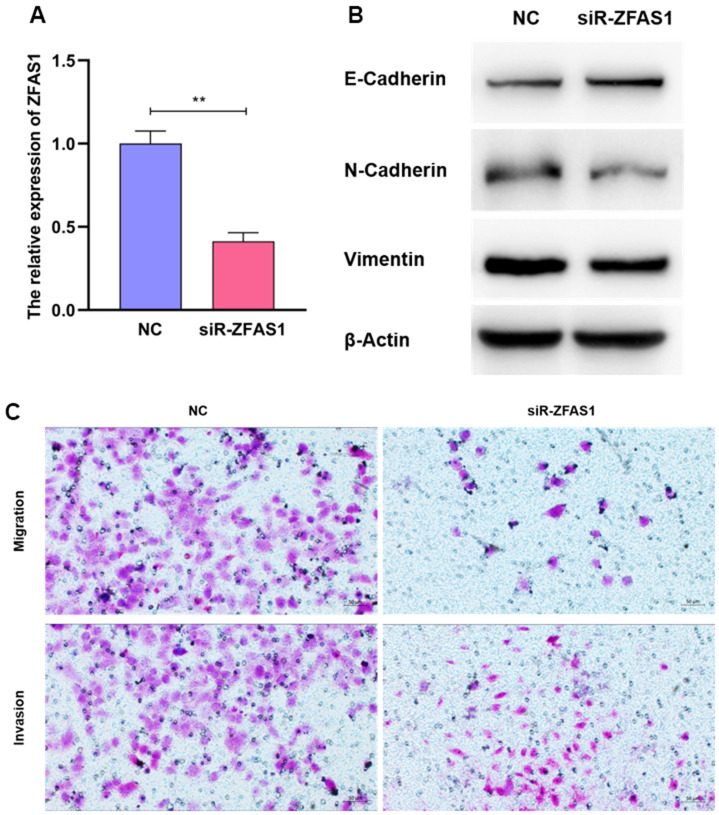
**ZFAS1 enhanced the abilities of invasion and migration of A549.** The knockdown efficiency of siR-ZFAS1 (**A**). Compared to the NC group, A549 cells transfected with siR-ZFAS1 showed higher expression of E-Cadherin and lower expression of N-Cadherin and Vimentin (**B**). SiR-ZFAS1 significantly reduced the ability of invasion and migration in A549 cell line (**C**).

To further validate the potency of ZFAS1 in regulating the metastatic process of LUAD, ZFAS1 was silenced using siRNA in A549 cell line with the highest expression (Figure 10A). Interestingly, we found that siRNA-ZFAS1 considerably decreased the invasion and migration abilities of A549 cell line, suggesting the pro-metastatic effect of ZFAS1 (Figure 10B). Consistently, siRNA-ZFAS1 provoked the remarkable reductions of VIMENTIN and NCADHERIN in the epithelial-mesenchymal transition pathway, but showing the opposite role to the E-CADHERIN (Figure 10C). These findings indicate that lncR-ZFAS1 plays a pivotal role in promoting LUAD metastasis.

## DISCUSSION

Efforts have long been made for the deeper understanding of LUAD metastasis which severely threatened survival [8, 16]. An increasing number of studies focused on the genetic profiling of tumor cells, which was prominently modulated by dynamic and complex alterations in the TME, and intended to dissect the metastatic mechanisms of LUAD [17–20]. Long non-coding RNAs have been well documented that play nonnegligible roles in regulating a diverse array of malignant processes of LUAD, including distant metastasis [21, 22]. For example, Chen et al. demonstrated that lncR-BC facilitated LUAD progression and metastasis through regulating the transcript variant and alternative splicing of a non-protein-coding inositol monophosphatase domain containing 1, besides, lncR-BC could interact with splicing factors, such as hnRNPK [23]. Another study of Hu revealed the pro-metastatic effect of lncRNAs in the TME of LUAD and they found that lncR00963 loaded in LUAD cell-derived extracellular vesicles fostered tumor growth and metastasis by facilitating Siah1 degradation but suppressing Zeb1 degradation [24].

In this study, we identified and validated that ZFAS1, SH3BP5-AS1 and AL359220.1 were metastasis-related and considerably regulated LUAD progression. In addition, we provided an accurate and promising MRRS based on the three metastatic lncRNAs for prognostic evaluation of LUAD. Lin et al. found that the elevation of SH3BP5-AS1 induced by N6-methyladenosine prominently facilitated pancreatic cancer cell migration by sponging miR-139-5p and targeting CTBP1, accordingly activating the WNT signaling pathway [25]. Besides, ZFAS1 has ever been demonstrated that closely affected the survival of patients with gastric cancer and could be served as a strong candidate for prognostic evaluation [26]. A study of Fan implied that increasing expression of ZFAS1 fostered LUAD progression by attracting more miR-1271-5p and upregulating FRS2 [27]. Nevertheless, few studies have revealed the potential role of AL359220.1 as yet.

Although these results demonstrated the potential of MRRS in assessing the metastatic possibility and prognostic status of LUAD patients and verified the significant relevance between ZFAS1 and clinicopathologic characteristics, some limitations remain to be further optimized. Exosomes containing a diverse array of cellular components and productions have been gradually documented that facilitate intercellular communication and modulate the genetic and functional alterations in cell populations in the TME [1, 28, 29]. However, how LUAD cell-derived exosomes regulate metastatic process and whether ZFAS1 is wrapped in exosomes and participates in the functional modulation in the TME of LUAD remain to be further investigated. Additionally, detailed molecular mechanisms by which ZFAS1 promotes LUAD metastasis are incompletely understood, so a series of *in vivo* and *in vitro* experimental models are needed to further decipher them from more novel and comprehensive perspectives. In the future, we will collect more clinical data from additional patients to perform machine learning and validate the effectiveness of our model.

## CONCLUSIONS

In this study, we identify and validate the metastatic relevance of sMRLNRs including AL359220.1, SH3BP5-AS1 and ZF-AS1, in various cell lines and tissues and elucidate their potential for prognostic assessment of LUAD by developing a MRRS. More importantly, we ascertain the close association of ZFAS1 with pathological stage and distant metastatic status of LUAD patients admitted to a single center. The above findings propose a novel link between sMRLNRs and LUAD metastasis, and help to open up a new perspective for metastasis-related studies.

## MATERIALS AND METHODS

### Sample collection and cell culture

One hundred and fifty-six LUAD tissues and adjacent normal tissues were collected from patients admitted to the First Affiliated Hospital of Chongqing Medical University between May 2018 and September 2022. All specimens were frozen in liquid nitrogen immediately and transferred to –80° C refrigerator for further analyses.

LUAC cell lines (A549, NCI-H1299 and HCC827) and human bronchial epithelial cell line (HBE) were purchased from the American Type Culture Collection (Manassas, VA, USA). DMEM and 1640 basic medium, supplementing with 10% fetal bovine serum, 100 μ/mL penicillin and streptomycin (Gibco, Gaithersburg, MD, USA) was utilized for cell culture. Cells were incubated at 37° C with an atmosphere of 5% CO_2_.

### Real-time quantitative PCR

According to the manufacturer's instructions, total RNA from tissues and cell lines under various experimental conditions was extracted by Triazole (Invitrogen, Waltham, MA, USA). cDNA Synthesis Kit (TaKaRa, Osaka, Japan) combining with RNA (1μg) was utilized to reverse transcribed cDNA. The quantitative polymerase chain reaction (qPCR) was performed on an ABI 7500 real-time PCR system (Applied Biosystems, Waltham, MA, USA) using SYBR-Green method (TaKaRa). Relative expression levels of lncRNAs normalized to GAPDH was calculated by the 2−ΔCt method. The primer sequences are shown in Table 3. Three assays were performed per cDNA sample.

**Table 3:** The sequences of ZFAS1, MCF2L-AS1, AL359220.1, GAPDH and siR-ZFAS1.

**ZFAS1**	F primer (5’-3’)	GCTATTGTCCTGCCCGTTAG
	R primer (5’-3’)	TCGTCAGGAGATCGAAGGTT
**MCF2L-AS1**	F primer (5’-3’)	GATCAACGTTCAATCCACCG
	R primer (5’-3’)	CGTCAAGATAGCGCAGCTTCC
**AL359220.1**	F primer (5’-3’)	TTGGGAGGGTGTGGGTATT
	R primer (5’-3’)	GGGACACCGCTGATCGTTTACCAAACCCRAAAACTACTC
**GAPDH**	F primer (5’-3’)	CTCTGCTCCTCCTGTTCGAC
	R primer (5’-3’)	ACCAAATCCGTTGACTCCGA
**SiR-ZFAS1**	Seq-1	CAAGGUUACUGUAUACAUAGC
	Seq-2	GAAUAUAUAUAUACAUAUAAA

Note: F primer, forward primer; R primer, reverse primer; Seq, sequence.

### Transcriptome data download and preprocessing

RNA-sequencing data and clinical data of LUAD patients were downloaded from the TCGA data portal (https://portal.gdc.cancer.gov/), containing 402 LUAD and 54 non-tumor samples (Supplementary Table 1). Our data were obtained from the TCGA database. We conducted preprocessing of the clinical data, excluding patients with a survival period of less than 30 days from the analysis. This exclusion was done because this subgroup of patients may have experienced early mortality due to factors such as bleeding and infection. These data were currently updated in Dec.12, 2022. Raw data were collected to do further analyses. RNA-seq results and clinical results were combined into a matrix file by a merge script of the Perl (http://www.perl.org/).

### MRLNRs extraction

The limma package of R software was used to screen differentially expressed lncRNAs (DElncRs) from TCGA dataset by comparing the LUAD and non-tumor samples. The screen criterion is as follows: P< 0.05 and |log2 fold change| > 1. MRGs were screened by The Molecular Signatures Database v4.0 (TOMIDA_METASTASIS_UP M17830, TOMIDA_METASTASIS_DN M2583, http://www.broadinstitute.org/gsea/msigdb/index.jsp). MRGs were used to establish the immune score of LUAD gene by GSEA. The correlations between immune score and the expression levels of lncRNAs in LUAD patients were calculated by Pearson correlation analysis. A standard of |r|>0.6 and P<0.01 was used to verify the MRLNRs.

### Acquire the survival-related MRLNRs (sMRLNRs)

MRLNRs with correlation of OS were regarded as sMRLNRs in LUAD patients. The sMRLNRs were screened by univariate COX analysis using R software survival packages (*P*<0.01). Besides, the sMRLNRs were divided into protective and deleterious portion by Hazard ratio. These sMRLNRs were used for subsequent research.

### Metastasis-related risk score model (MRRS) creation

The sMRLNRs have been analyzed by multivariate COX analysis, and the integrated sMRLNRs were utilized as an independent prognostic factor to develop the MRRS (*P*<0.05). We performed the MRRS to classify LUAD patients into the high-risk group and the low-risk group.

The creation of MRRS was based on the expression data multiplied by Cox regression coefficients. The formula was as follows, [Expression levels of AL359220.1 * (-0.27974)] + [Expression levels of SH3BP5-AS1 * (-0.16829)] + [Expression levels of ZF-AS1 * (0.142708)]. Patients were divided into high-risk group and low-risk group according to the median score of the MRRS.

### Bioinformatics analysis

Kaplan-Meier curve was used to evaluate the OS between high-risk group and low-risk group. Univariate Cox regression analysis and Pearson correlation analysis were used to identify the interest MRLNRs. Univariate and multivariate Cox regression analysis were used for identify the independent prognostic factors of LUAD patients. Based on the MRRS, ROC curve was employed to assess the sensitivity and specificity of the prognosis. Gene set enrichment analysis (GSEA) was displayed to explore the different functional phenotypes between the high-risk group and the low-risk group. The nomogram was constructed by rms package of R software to provide a reference for clinical evaluation of LUAD patients’ prognosis.

### Statistical analysis

SPSS21.0 software (SPSS Inc, Chicago, IL, USA) and GraphPad Prism5 (GraphPad Software Inc, La Jolla, CA, USA) were employed to analyze data. Data were expressed as means ± SD. The relevance between lnR-ZFAS1 and clinicopathological features of LUAD patients was detected by using Fisher’s exact probability method. The differential comparison between two or more groups were analysed through Student T-test or ANOVA and post-hoc test respectively. *P*<0.05 was considered a significantly statistical difference.

### Availability of data and materials

Authors can provide all of the datasets on reasonable request.

## Supplementary Material

Supplementary Table 1
