# Supplementary material for: Metastasis-related long non-coding RNAs AL359220.1, SH3BP5-AS1 and ZF-AS1 are significant for prognostic assessment of lung adenocarcinoma
Source: Aging (Albany NY). 2023 Aug 10;15(15):7551–64. doi: 10.18632/aging.204923 (PMC10457074; doi:10.18632/aging.204923)
Supplement: Supplementary Table 1 [file aging-15-204923-s001.pdf]

SUPPLEMENTARY TABLE

Supplementary Table 1. Basic clinical characteristic information of LUAD patients from TCGA.

Characteristic		TCGA
**Age**	<65	203 (45%)
	≥65	253 (55%)
**Gender**	FEMALE	248 (54%)
	MALE	208 (46%)
**Stage**	Stage I-II	356 (78%)
	Stage III-IV	100 (22%)
**T stage**	T1-2	397 (87%)
	T3-4	59 (13%)
**N stage**	N0	304 (67%)
	N1-3	152 (33%)
**M stage**	M0	435 (95%)
	M1	21 (5%)
